# ED_50_ and ED_95_ of hypobaric ropivacaine during unilateral spinal anesthesia in older patients undergoing hip replacement surgery

**DOI:** 10.3389/fmed.2025.1571574

**Published:** 2025-07-24

**Authors:** Chao Lin, Wen-Lin Xian, Jun Xu, Ting Zhao, Zhi-Qiang Wu, Fang-Jun Wang

**Affiliations:** ^1^Department of Anesthesiology, Affiliated Hospital of North Sichuan Medical College, Nanchong, China; ^2^Department of Anesthesiology, People's Hospital of Yilong County, Nanchong, China

**Keywords:** hypobaric ropivacaine, unilateral spinal anesthesia, geriatrics, hip arthroplasty, dose-response relationship

## Abstract

**Objective:**

The spinal block was limited to the operative side during unilateral spinal anesthesia, which has less physiological interference and fewer complications for the patient. The optimal dose of ropivacaine for unilateral spinal anesthesia is still unclear. The aim of this trial was to investigate the ED_50_ and ED_95_ of hypobaric ropivacaine during unilateral spinal anesthesia in older patients undergoing hip arthroplasty.

**Methods:**

All patients were administered hypobaric ropivacaine at a spinal anesthetic drug concentration of 0.4%. The trial was conducted using the Dixon sequential method with an initial dose of 10 mg of ropivacaine and a dose difference of 0.5 mg between two consecutive patients. If the subject’s post-block non-surgical leg Bromage score is greater than 0, the next subject’s dose of ropivacaine is reduced by 0.5 mg. If the subject’s post-block non-surgical leg Bromage score is equal to 0, the next patient’s dose of ropivacaine is increased by 0.5 mg. The trial was terminated when alternating positive–negative results were obtained for 7 pairs of patients. ED_50_, ED_95_, and the corresponding 95% confidence intervals (CI) for unilateral spinal anesthesia with 0.4% hypobaric ropivacaine were calculated using probit regression analysis. Patients with motor block in the non-surgical leg were identified as the positive group, and patients without motor block in the non-surgical leg were identified as the negative group. Anesthesia onset time, sensory block duration, duration of motor block, the highest dermatomes blocked on the surgical side, and postoperative complications were compared between the two groups.

**Results:**

The ED_50_ and ED_95_ of hypobaric ropivacaine during unilateral spinal anesthesia in older patients undergoing hip arthroplasty were 11.13 mg (95% CI: 10.85–11.42 mg) and 10.30 mg (95% CI: 9.04–10.65 mg), respectively. The drug dosage was higher in the positive group than in the negative group. There was no differences in the onset of anesthesia, block plane of the affected side, the sensory and motor block time of affected side, and the incidences of low blood pressure, nausea and vomiting, chills, urinary retention between groups (*p* > 0.05).

**Conclusion:**

The ED_50_ and ED_95_ of hypobaric ropivacaine during unilateral spinal anesthesia in older patients undergoing hip arthroplasty were 11.13 mg (95% CI: 10.85–11.42 mg) and 10.30 mg (95% CI: 9.04–10.65 mg), respectively. Unilateral spinal anesthesia limits the level of block to the operative side with less physiologically disruptive to the patient, more stable perioperative hemodynamics and fewer complications.

## 1 Introduction

The number of older patients with hip diseases such as femoral neck fracture or femoral head necrosis has gradually increased in recent years, and hip arthroplasty is still a widely used and effective treatment for patients with hip diseases. However, the older patients have reduced physiologic reserve, often combined with a variety of associated diseases, a reduced tolerance to surgery and a decreased ability to regulate stress, which increases the incidence of postoperative complications and the mortality (1, 2). The anesthesia modalities used currently for older patients undergoing hip arthroplasty are general anesthesia, neuraxial anesthesia (spinal anesthesia and epidural anesthesia), and nerve blocks (3). Older patients undergoing hip arthroplasty under spinal anesthesia have lower postoperative complications and mortality compared to patients undergoing general anesthesia. Delayed recovery of sensory-motor function and prolonged stay in PACU often occur in patients undergoing spinal anesthesia after surgery (4). In addition, the incidence of respiratory depression, hypotension, nausea and vomiting, and urinary retention is increased when spinal anesthesia block is too high and produces extensive sympathetic blockade (5, 6).

Most lower extremity surgeries involve only one side, and conventional spinal anesthesia inevitably causes blockage on the healthy side. However, unilateral spinal anesthesia limits the level of blockage to the operative side, which has less physiologically disruptive to the patient (7). It has been shown that the administration of unilateral spinal anesthesia can reduce the frequency and severity of intraoperative hypotension in patients, decrease the use of vasoactive medications, and shorten the duration of surgery compared to the general anesthesia (1). Previous studies have found that the administration of excessive drug dosage can induce the blockade of the non-surgical leg occurs during unilateral spinal anesthesia (8). It has also been reported that during unilateral spinal anesthesia, some patients have incomplete block on the surgical side, or the duration of block is too short to meet the surgical needs, etc. (1). Although the ED50 of 0.5% hypobaric ropivacaine has been studied in the past (9), its anesthesia was effectively defined as the T10 sensory blockade level was maintained for more than 60 min, and the Bromage score of the surgical side reached 3 within 10 min after the administration of the drug. The blockade of the non-surgical leg was not observed, and there was no guarantee that the non-surgical leg would not be blocked. Moreover, the maximum dose of hypobaric ropivacaine for unilateral spinal anesthesia is unclear. Therefore, it is necessary to determine the optimal dose of hypobaric ropivacaine, which can provide the longest effective blockade of surgical limbs without blocking non-surgical leg.

The aim of this trial was to investigate the ED_50_ and ED_95_ of hypobaric ropivacaine for unilateral spinal anesthesian in older patients undergoing hip arthroplasty, and to provide clinical guidance for the administration of unilateral spinal anesthesia.

### 1.1 Information and methods

#### 1.1.1 General information

The study was approved by the Ethics Committee of the Affiliated Hospital of North Sichuan Medical College (2024ER330-1) and registered in the China Clinical Trials Registry on July 11, 2024 (www.chictr.org.cn/showproj.html?proj=233031; Registration No.: ChiCTR2400086829). All procedures in this trial followed the Declaration of Helsinki, and all patients signed the informed consent for clinical trials and the informed consent for anesthesia. Inclusion criteria: (1) ASA classification I ~ II; (2) patients undergoing elective hip replacement surgery; (3) age ≥ 65 years old; (4) BMI 18.5 ~ 30 kg/m^2^. Exclusion criteria: (1) History of spinal surgery, spinal deformity; (2) History of allergies to ropivacaine; (3) Platelet count <80 × 10^9^ / L, recently on anticoagulant medication or coagulation disorders; (4) Cardiac, hepatic or renal insufficiency; (5) Persons with motor dysfunction of the healthy lower limb; (6) Speech communication disorder; (7) Those who cannot perform anesthesia in the lateral position; (8) Puncture site infection. Withdrawal criteria: (1) Serious hemodynamic fluctuations during the clinical study endangered the patient’s life and safety; (2) Change of the surgical plan; (3) Failure of neuraxial anesthesia procedure; (4) Patients and their families requested to withdraw from the experiment.

## 2 Methods

All patients were fasted for 8 h and abstained from drinking for 2 h preoperatively without preoperative medication. After arriving at the operating room, patientswere routinely monitored with heart rate (HR), electrocardiogram (ECG), noninvasive blood pressure (NBP), and peripheral pulse oximetry (SpO2). Oxygen was administered by mask at 6 L/min, and intravenous infusion of compound sodium chloride solution was at a rate of 8 ~ 10 mL kg^−1^-h^−1^. 0.4% hypobaric ropivacaine was prepared by a nurse anesthetist who was not involved in the assessment. Dosing method for 0.4% hypobaric ropivacaine: 4 mL of 1% ropivacaine plus 6 mL of sterilized water for injection is configured to 10 mL. The patient was placed in the lateral position with the affected side facing upward and the L2-3 intervertebral space was selected as the puncture point. The epidural puncture needle was used in a straight-in puncture, and the disappearance of resistance method was used to determine whether the epidural puncture needle successfully entered the epidural space. Then 0.4% hypobaric ropivacaine was administered at a rate of 0.1 mL/s by the needle-through-needle technique, and the spinal anesthesia needle port was angled upward at 45° from the level of the spine (10). After withdrawing the spinal anesthesia needle, the catheter was placed into the epidural space 4–5 cm for backup. The initial dose of 0.4% hypobaric ropivacaine was determined to be 10 mg based on pre-tests. The trial utilized a Dixon sequential method, where the subject’s dose of ropivacaine depended on the modified Bromage score of the previous patient’s non-surgical leg. When motor block was present at a Bromage score greater than 0 in the non-surgical leg (positive response), the dose of ropivacaine in the next patient was reduced by 0.5 mg, and vice versa (negative response), the dose of the drug was increased by 0.5 mg. The test was ended when there are 7 positive–negative turning points. According to the results of published studies (11), 7 turning points may occur in 20–30 patients. In order to make the experimental results more realistic and reliable, the sample size of this study was appropriately increased to include a total of 40 patients. With the Dixon sequential method, there is a positive response where the non-surgical leg is blocked and a negative response where the non-surgical leg is not blocked. Therefore, in this study, patients with positive reactions were identified as the positive group and those with negative reactions were identified as the negative group. Differences between the two groups were compared in the onset time of anesthesia, the highest dermatomes blocked, the sensory block time, the motor block time of the lower limb on the surgical side, and postoperative complications.

### 2.1 Scoring criteria

A modified Bromage score was used to assess the patients’ motor block in both lower limbs: 0 = no motor block; 1 = inability to lift the leg; 2 = inability to bend the knee; 3 = inability to bend the ankle (12). Bilateral motor block was assessed every minute for the first 15 min after subarachnoid injection of ropivacaine, then it was evaluated every 10 min until the modified Bromage score of the lower limb on the surgical side was 0. The plane of anesthetic block (nociception) was determined using the pinprick test (13).

### 2.2 Observation indexes

The primary outcome was the ED_50_ and ED_95_ of 0.4% hypobaric ropivacaine for unilateral spinal anesthesia in geriatric hip arthroplasty. The secondary outcomes were the time of onset and regression of motor block on the surgical side, the highest dermatomes blocked and duration of sensory block on the surgical side, and Bromage motor block scores both on the surgical side and non-surgical leg, incidence of bradycardia and hypotension, duration of surgery, Intraoperative fluid replacement, intraoperative bleeding, and the occurrence of postoperative adverse reactions (urinary retention, chills, dizziness, headache, nausea and vomiting) recorded 24 h postoperatively.

### 2.3 Statistical analysis

Statistical analysis was performed using SPSS27.0 software. Measurements were expressed as mean ± standard deviation (SD) and *t*-test was used for comparison between groups. Count data were expressed as relative numbers, and the 2 test was used between groups. A probit regression analysis was used to calculate the ED_50_, ED_95_, and corresponding 95% confidence intervals (CIs) for unilateral spinal numbness produced by 0.4% hypobaric ropivacaine. Plotting of hypobaric ropivaca sequence diagrams and dose-effect relationship curves. Differences were considered statistically significant at *p* < 0.05.

## 3 Results

**Figure 1:**
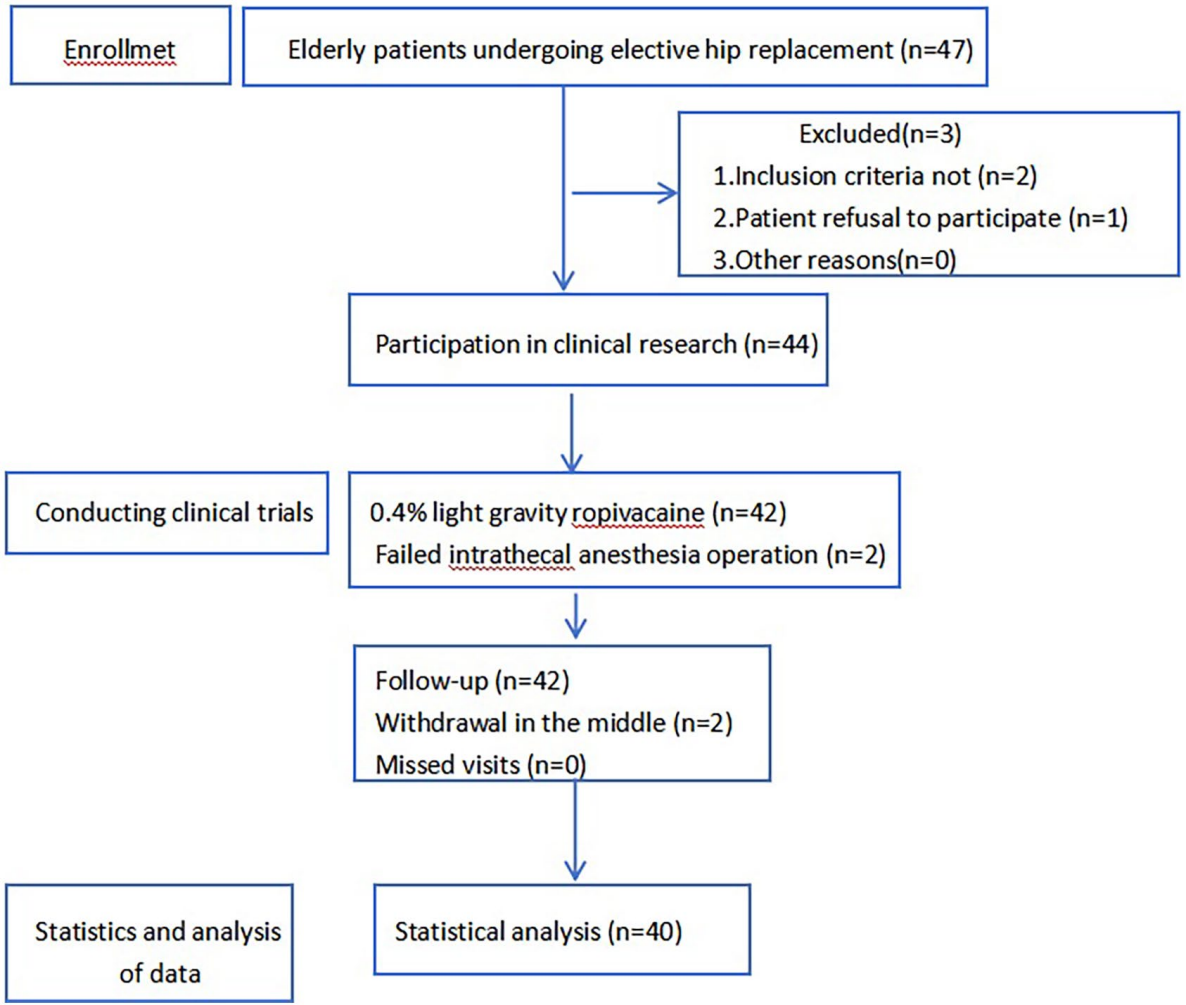
Participant flow diagram.

A total of 47 patients were recruited for this study, of whom 3 did not meet the inclusion criteria, 2 failed intrathecal anesthesia operations, and 2 withdrew from the study midway. A total of 40 subjects’ data participated in the statistical analysis of the data (Figure 1), including 21 cases in the negative group and 19 cases in the positive group.

### 3.1 Comparison of the demographic characteristics of patients in the two groups

There was no statistically significant difference between the two groups in terms of gender, age, height, weight, ASA classification, surgical time, intraoperative IV fluid volume, and intraoperative bleeding (*p* > 0.05), as shown in Table 1.

**Table 1:** The demographic data of the patients in the two groups.

Index	Negative group (21)	Positive group (19)	χ^2^/t	*p*
Gender, *n* (%)				
Male	11 (52.4)	7 (36.8)	0.973	0.324
Female	10 (47.6)	12 (63.2)		
Age, mean (SD) (years)	69.6 (3.5)	71.6 (4.0)	1.689	0.099
Height, mean (SD) (cm)	163.6 (7.4)	161.6 (7.8)	0.827	0.414
Weight, mean (SD) (kg)	67.4 (8.2)	65.3 (7.1)	0.865	0.393
ASA grade, *n* (%)				
I	6 (28.6)	5 (26.3)	0.025	0.873
II	15 (71.4)	14 (73.7)		
Surgical time, mean (SD) (min)	63.80 (8.84)	65.68 (9.94)	0.631	0.532
Intraoperative IV fluid volume, mean (SD) (ml)	733.8 (86.2)	764.2 (107.2)	0.993	0.327
Intraoperative bleeding, mean (SD) (ml)	223.7 (72.2)	256.1 (99.2)	1.189	0.242

ASA, American Society of Anesthesiologists; SD, standard deviation.

### 3.2 The ED_50_, ED_95_ and dose-effect curve of hypobaric ropivacaine for unilateral spinal anesthesia

**Figure 2:**
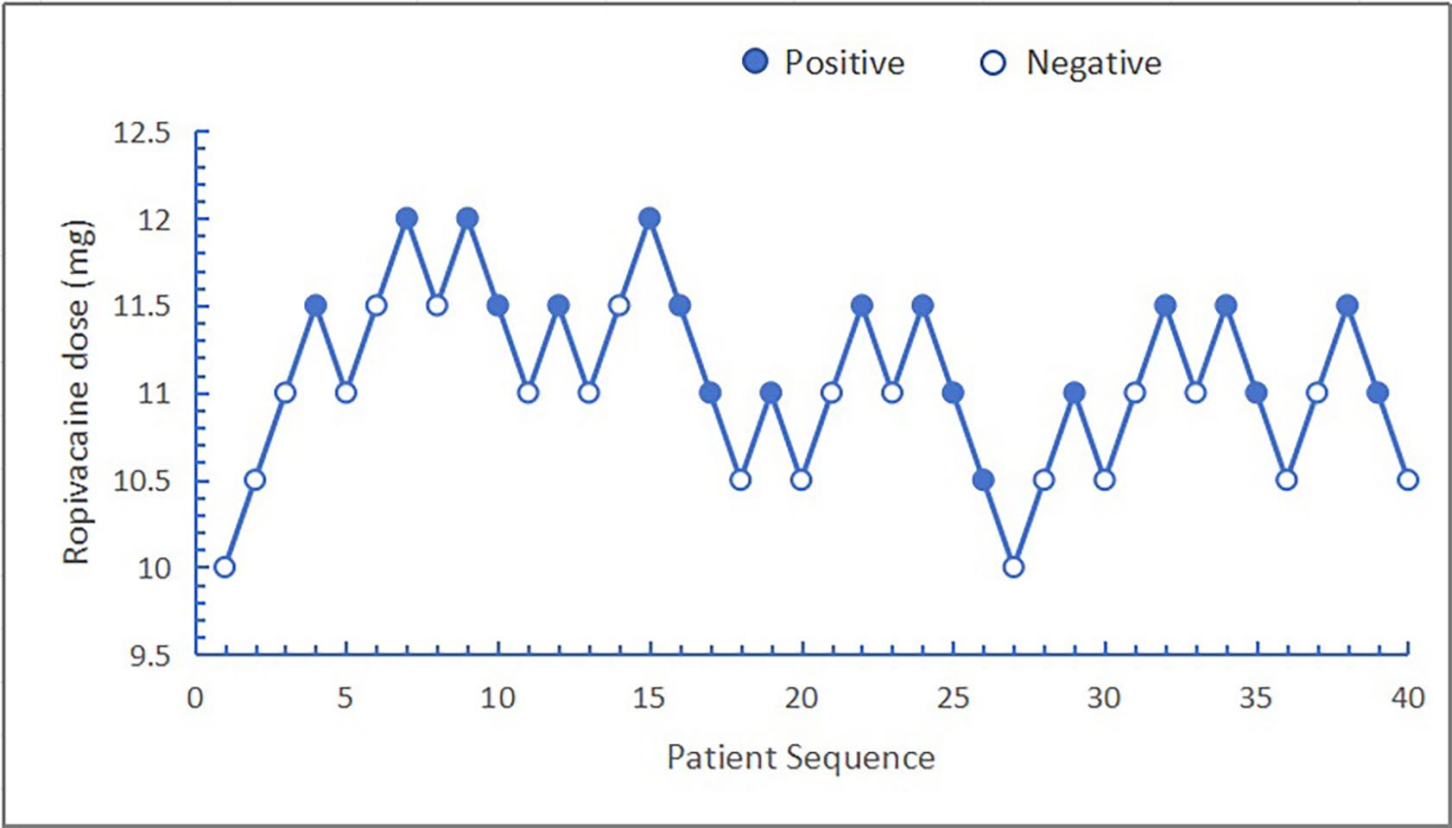
Sequence diagram of hypobaric ropivacaine for unilateral spinal anesthesia in the older patients undergoing hip arthroplasty.

**Figure 3:**
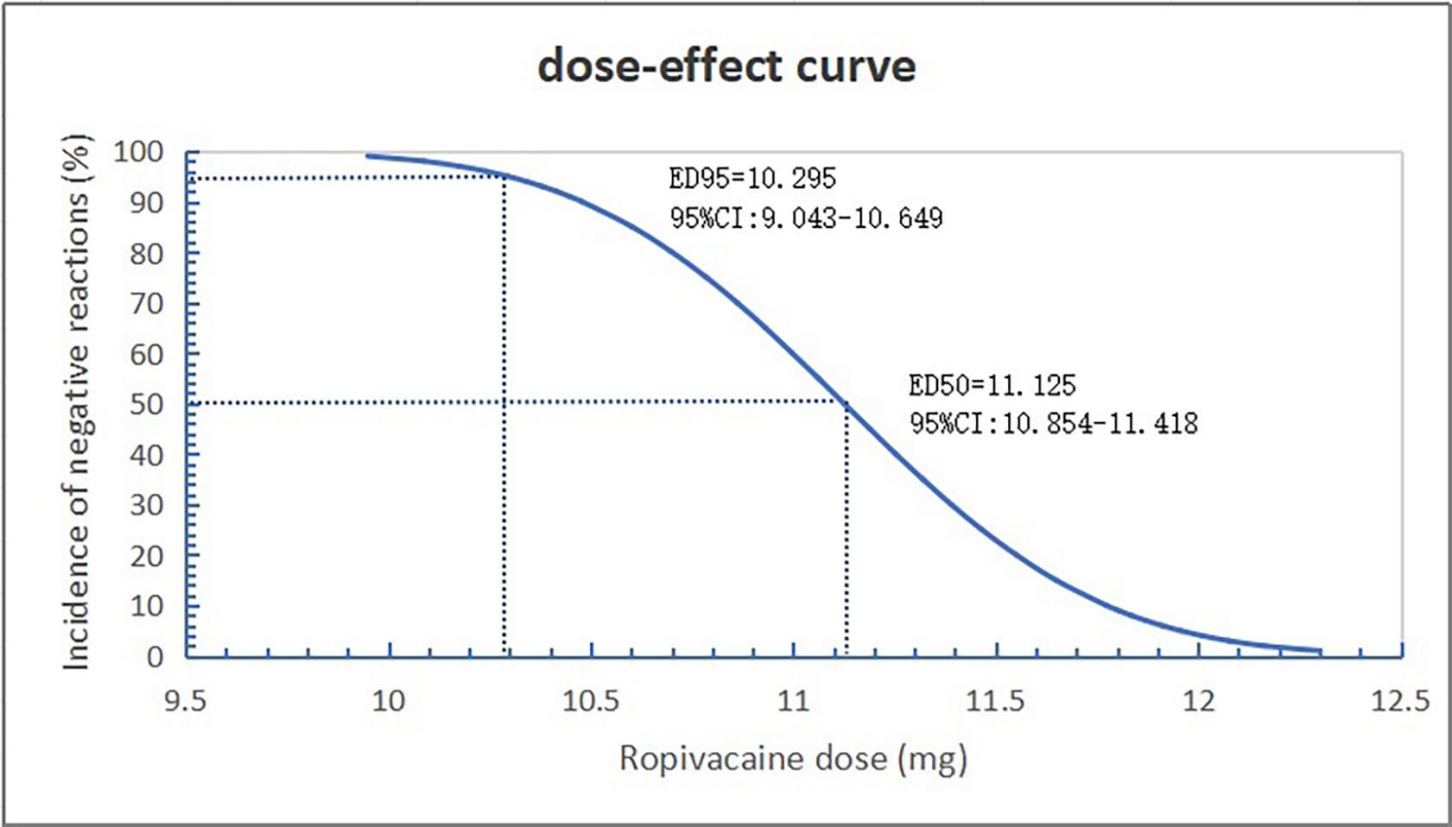
Dose-effect curve of hypobaric ropivacaine for unilateral spinal anesthesia in geriatric hip arthroplasty.

The ED_50_ and ED_95_ corresponding doses of hypobaric ropivacaine for unilateral spinal anesthesia in geriatric hip arthroplasty were calculated from probabilistic regression analyses to be 11.13 mg (95% CI: 10.85–11.42 mg) and 10.30 mg (95% CI: 9.04–10.65 mg), respectively (Figures 2, 3).

### 3.3 Anesthesia characteristics and complications

There was a statistical difference in ropivacaine dosage between the two groups (*p* < 0.05). There were no statistically significant differences in the onset time of anesthesia, the highest dermatomes blocked, the sensory block time, and the motor block time of the lower limb on the surgical side (*p* > 0.05). There was no statistical difference in the incidence of hypotension, nausea and vomiting, chills, and urinary retention (*p* > 0.05). No bradycardia, respiratory depression, or headache complications were observed in either group (Tables 2, 3).

**Table 2:** Anesthesia characteristics in the two groups.

Index	Negative group *n* = 21	Positive group *n* = 19	χ^2^/t	*p*
Ropivacaine dose, mean (SD) (mg)	10.81 (0.43)	11.37 (0.40)^*^	4.217	<0.001
Anesthesia onset time, mean (SD) (min)	7.62 (1.24)	7.26 (1.19)	0.921	0.363
Sensory block duration, mean (SD) (min)	133.23 ± 15.27	138.63 ± 18.86	0.998	0.325
Duration of motor block, mean (SD) (min)	113.33 ± 15.17	115.68 ± 18.46	0.442	0.661
The highest dermatomes blocked on the surgical side, *n* (%)				
T4	1 (4.8)	4 (21.1)	3.632	0.163
T6	11 (52.4)	11 (57.9)		
T8	9 (42.9)	4 (21.1)		

Compared to the negative group, ^*^p < 0.05.

**Table 3:** Complications in the two groups.

Index	Negative group *n* = 21	Positive group *n* = 19	*χ* ^2^	*p*
Hypotension, *n* (%)	1 (4.8)	4 (21.1)	2.420	0.120
Bradycardia, *n* (%)	0 (0)	0 (0)	—	—
Nausea and vomiting, *n* (%)	1 (4.8)	3 (15.8)	1.348	0.246
Respiratory depression, *n* (%)	0 (0)	0 (0)	—	—
Chills, *n* (%)	2 (9.5)	4 (21.1)	1.040	0.308
Headache, *n* (%)	0 (0)	0 (0)	—	—
Urinary retention, *n* (%)	1 (4.8)	3 (15.8)	1.348	0.246

## 4 Discussion

In this study, the ED_50_ and ED_95_ of 0.4% hypobaric ropivacaine for unilateral spinal anesthesia in older patients undergoing hip arthroplasty were 11.13 mg (95% CI: 10.85–11.42 mg) and 10.30 mg (95% CI: 9.04–10.65 mg), respectively. Compared with conventional spinal anesthesia in previous studies (14, 15), it can reduce intraoperative hemodynamic fluctuations in patients, and reduce the incidence of complications such as urinary retention, nausea and vomiting. It is a safe and effective anesthesia strategy.

The results of this study showed that the dose of 0.4% ropivacaine in the positive group was significantly higher than that in the negative group, indicating that the same concentration of hypobaric ropivacaine used in hip arthroplasty in older patients increases the probability of the patient producing bilateral spinal anesthesia as the dose of the drug increases. In a previous study (9), Wang Weibing et al. found that the ED_50_ of 0.5% hypobaric ropivacaine to meet the surgical needs of older patients undergoing hip arthroplasty was 6.43 mg, and their observation indexes were the sensory and motor blockage on the affected side, and they did not observe the blockage on the healthy side, which may not be the maximum ED_50_ of ropivacaine that produces unilateral spinal. In this study, the median effective concentration of 0.4% hypobaric ropivacaine to produce unilateral spinal anesthesia was 11.13 mg by the sequential method, observing the patients’ motor block on the healthy side. Compared with the results of the present study, the time of onset of anesthesia was the same for both, but there were large differences in the highest dermatomes blocked on the surgical side and the duration of sensory and motor blockade. Although the drug concentration of 0.5% used by Weibing Wang et al. was higher than the 0.4% used in the this study, the median effective concentration of 11.13 mg in the present trial was much higher than that of 6.43 mg in terms of the dosage administered. This is mainly because we studied the maximum dose of hypobaric ropivacaine to produce unilateral spinal anesthesia drug. Also in their study, the results showed that the majority of hip arthroplasty patients require additional drug administration through an epidural catheter to meet the needs of the procedure. In our study, all surgical procedures were performed under unilateral spinal anesthesia without the need for additional epidural administration, indicating that unilateral spinal anesthesia can meet the needs of lower extremity orthopedic surgical operations. It was shown that 10.30 mg of hypobaric ropivacaine for unilateral spinal anesthesia enables elderly patients to meet the demands of orthopedic surgical manipulation of the lower extremities while producing only the block of the affected lower extremity.

In the comparison of the onset of anesthesia between the two groups, although the negative group (7.62 min) was longer than the positive group (7.26 min), the difference was not statistically significant. The onset of anesthesia with 0.75% ropivacaine and 0.5% bupivacaine was 6.73 min and 5.12 min, respectively, in previous studies (16), suggesting that the onset of local anesthetics is mainly related to the drug’s own pharmacological characteristics. The onset time of ropivacaine at 0.75% in previous studies (16) was shorter than the onset time of ropivacaine at 0.4% in the present study, so when ropivacaine is used for spinal anesthesia, it is possible that the onset time of anesthesia may be shortened as the concentration of the drug increases. However, further validation is needed in the future in the same type of surgery at the same drug dose. There was no statistically significant difference in the anesthetic block plane between the two groups, but more patients in the positive group had a block plane at the T4 level. This may be related to the increase in the plane of anesthetic block as the dose of the drug increases. There was no statistically significant difference in the duration of sensory and motor block in the negative and positive groups in this study, but both were more than 2 hours, which is able to meet the needs of most lower extremity surgeries such as hip arthroplasty, knee arthroplasty, and saphenous vein (17–19).

No bradycardia, respiratory depression, or headache complications were observed in either group of patients in this study. Cicekci F et al. (20) in comparing spinal anesthesia and unilateral spinal anesthesia found that the difference in the incidence of hypotension and urinary retention between the two groups was higher in the spinal anesthesia group although it was not statistically significant, which is consistent with the results of our study. The incidence of nausea and vomiting and chills in the positive group (15.8 and 21.1%) was significantly higher than that in the negative group (4.8 and 9.5%) in this study, but the difference was not statistically significant. There may be two reasons for this outcome. on the one hand, it may be that the sample size is small enough to produce statistical differences. On the other hand, the reason may be that even though bilateral lower limb blocks occurred in the positive group, it was still not a routine spinal anesthesia, and therefore some patients did not have the corresponding complications.

Unilateral spinal anesthesia requires a certain amount of time in lateral recumbency for the purpose of blocking only the surgical side (21, 22). A bilateral lower extremity block occurs when the patient’s position is changed before the time in lateral recumbency has reached adequate binding of ropivacaine to a unilateral spinal nerve. The test subjects in this study were hip arthroplasty patients and the surgical position was the lateral position. The patients were kept in lateral position from the beginning of anesthesia till the end of the surgery, so there was no influence of surgical position on the outcome of the study. To increase the applicability of the findings, the BMI of the study population in this trial was 18.5 ~ 30 kg/m2. However, previous studies have found that pregnant women with different BMIs have similar dosage requirements for ropivacaine (23), so the BMI of the patients in this study would not have affected the results. The study population selected for this study was elderly patients with ASA I or II, therefore the dose of 10.30 mg for this study’s outcome, ED95, may not be applicable to patients with other health conditions (ASA III or IV) and other age groups. Further studies in this area are needed in the future.

Unilateral spinal anesthesia aims to limit the distribution of the spinal anesthetic block to the operative side, which is achieved by intrathecal injection of a non-isotonic local anesthetic drug and maintaining the lateral position for 15–20 min (24). Unilateral spinal anesthesia can be achieved by using small doses of local anesthetic drugs to limit bilateral blocks, rapidly restore motor function, and reduce the incidence of adverse cardiovascular effects. It also reduces stress at the time of surgery thereby improving surgical outcomes, minimizing complications, and greatly reducing recovery time (25, 26). Since the distance between the right and left nerve roots of the lumbar and thoracic spine is about 10–15 mm, when the appropriate amount of hypobaric local anesthetic drug is injected into the sheath while the patient is kept in the lateral position, so that the drug is fully combined with the superior side of the nerves, the nerve block can be restricted to one side and a unilateral block can be produced (15). Thus, unilateral spinal anesthesia allows for adequate motor and sensory blockade on the patient’s operative side, providing the surgeon with excellent surgical conditions. It also avoids unnecessary blockage of the non-surgical leg, resulting in less physiological interference with the patient and a better patient experience.

Our study has limitations. First, in this study, a small number of patients in the negative response group had their sensory nerves blocked on the healthy side, and one of them experienced urinary retention. Further clinical studies are needed to confirm whether the occurrence of urinary retention in patients with unilateral spinal anesthesia is related to the blockade of the sensory nerves on the healthy side. Second, although the incidence of postoperative complications in the positive group was higher than that in the negative group in this study, the difference was not statistically significant, which may be due to the small sample size, and further increase in the sample size is needed in the future to prove this result. Third, since hip arthroplasty requires patients to remain in the lateral position during the operation, the duration of unilateral spinal anesthesia plane immobilization was not studied, and further studies on the duration of unilateral spinal anesthesia plane immobilization are needed in the future. Fourth, in this study, only the drug dose of hypobaric ropivacaine to produce unilateral spinal anesthesia was measured, and further studies on the drug dose of hyperbaric ropivacaine and other spinal anesthetics with different gravity are needed in the future. Finally, the drug dosage in this study was only applicable to elderly patients with ASA I or II, and further research is needed in the future to investigate the drug dosage of hypobaric ropivacaine to produce unilateral spinal anesthesia in patients with other health statuses (ASA III or IV) and in other age groups.

In conclusion, the ED_50_ and ED_95_ of 0.4% hypobaric ropivacaine for unilateral spinal anesthesia in older patients undergoing hip arthroplasty were 11.13 mg (95% CI: 10.85–11.42 mg) and 10.30 mg (95% CI: 9.04–10.65 mg), respectively. The recommendation of 10.30 mg of hypobaric ropivacaine for unilateral spinal anesthesia allows elderly patients to meet the requirements of orthopedic surgical manipulation of the lower extremities while blocking only the affected lower extremity. Unilateral spinal anesthesia limits the level of block to the operative side, which is less physiologically disruptive to the patient, resulting in more stable perioperative hemodynamics and fewer complications.

## Data Availability

The datasets presented in this study can be found in online repositories. The names of the repository/repositories and accession number(s) can be found in the article/supplementary material.
